# Isolation of T cells from mouse oral tissues

**DOI:** 10.1186/1480-9222-16-4

**Published:** 2014-03-10

**Authors:** Pushpa Pandiyan, Natarajan Bhaskaran, Yifan Zhang, Aaron Weinberg

**Affiliations:** 1 Department of Biological Sciences, School of Dental Medicine, Case Western Reserve University, Cleveland, OH 44106, USA

**Keywords:** Murine oral tissue, Leukocyte isolation and oral T cells, T_reg_, Th17, ILC

## Abstract

**Background:**

Utilizing mouse models provides excellent immunological and experimental tools to study oral immune responses. However for functional assays, isolating T lymphocytes from the oral tissues has proved to be challenging due to the absence of reliable methods that yield viable cells with consistency. To study adaptive immune cell interactions in the oral mucosal tissues, it is necessary to isolate T cells with a good viability and study them at the single cell level.

**Findings:**

We have established an improved method to isolate immune cells, including T_regs_ and Th17 cells from intra-epithelial niches and lamina propria of the tongue, gingival and palatal tissues in the oral mucosa of mice.

**Conclusion:**

This new method of isolating immune cells from oral tissues will enable us to further our understanding of oral tissue immune cells and their role during oral infections and oral inflammation.

## Findings

Oral immunity and inflammation are associated with a wide array of human health conditions including, but not limited to, cancer, cardiovascular disease, graft versus host disease (GVHD), and infectious diseases such as AIDS [1-5]. To study infections and inflammation, murine models may be employed and are excellent tools of immunology [6,7]. Cells such as T cells, dendritic cells, and monocytes are major components of the adaptive immune system and these types of cells have been studied extensively in peripheral immune organs and mucosal tissues, such as the gut and skin. Yet, these types of immune cells that populate the oral mucosal tissues have been studied much less and their precise functions in the oral mucosa remain unclear. For example, studies have focused on T cells which are present in intra-epithelial compartments; i.e., oral lymphoid foci, and lamina propria [8-10,17], and determined that these cells play significant roles in oral immunity and inflammation [7,11,12]. The majority of these studies have examined the oral tissues using manual *in situ* immunohistochemistry or immunofluorescence microscopy [9,10]. While these studies have provided us basic information about these T cells, these methods do not provide us with clear information regarding their functions. Although T cells have been studied in oral tissues by flow cytometry [13], the cells isolated using such crude methods frequently show poor viability. It is difficult to study them *in vitro*, due to the scarcity and inconsistent viability of these cells. Recently, a protocol was published to detect T cells from the gingival tissues [14], but not from the tongue and palatal tissues. Here we describe a new, reproducible protocol to isolate > 94% viable leukocytes from mouse oral palatal and tongue tissues.

The oral palatal, sublingual and tongue tissues are dissected by incising the joint on both sides of the mouth, opening the jaws and exposing the mouth [15]. The tissues from two mice are collected in ice-cold phosphate buffered saline (PBS) with antibiotics (penicillin (100 U/ml) and streptomycin (100 U/ml). The tissue pieces are flushed twice with PBS containing antibiotics; and are cut into 3 mm × 3 mm pieces of tissues. The pieces are transferred to a 50 ml conical tube and rinsed 3 times with 2 ml of ice-cold RPMI-1640 containing antibiotics, 3% fetal bovine serum (FBS) and 20 mM HEPES, followed by decanting of the supernatant. The epithelium is disrupted by adding 10 ml of RPMI-1640 containing antibiotics, 3% FBS, 5 mM EDTA, 1 mM DTT and 20 mM HEPES to the tissue pieces and incubating for 20 minutes at 37°C. The pieces are allowed to sediment and the supernatant containing epithelial cells and intra-epithelial lymphocytes is strained through a 70 μm cell strainer into a fresh 50 ml tube. The flow through is then kept separately on ice (Fraction-1). The epithelial cells and intra-epithelial lymphocytes within the sedimented tissues are further removed by intense vortexing in 5 ml of RPMI-1640 containing antibiotics, 2 mM EDTA and 20 mM HEPES. The supernatant is collected and strained using a 100 μm strainer into another 50 ml tube and kept on ice (Fraction-2). The process is repeated twice. Leaving the tissue pieces behind, the supernatant is collected and strained again into the same Fraction-2 tube on ice. The fraction-2 supernatant will be a mixture of intra-epithelial leukocytes and epithelial cells and has to be further separated by gradient centrifugation. The tissue pieces are rinsed 2-3 times with 5 ml ice cold PBS to remove EDTA, decanting the supernatant into the Fraction-2 tube. The sedimented fragments are diced into smaller pieces and are added to a 15 ml tube with 1 ml of RPMI-1640 with antibiotics, collagenase-H (0.5 mg/ml), DNase (0.5 mg/ml) and 20 mM HEPES, and incubated at 37°C for 15 minutes (Collagenase-H, SIGMA (34 Units/mg). After 15 minutes, another 1 ml of collagenase buffer is added and incubated for an additional 15 minutes. The resulting viscous solution is vortexed intensely and strained using a 70 μm strainer into a 50 ml tube on ice (Fraction 3). 10 ml of RPMI-1640 containing antibiotics, 3% FBS, DNase (0.5 mg/mL) and 20 mM HEPES is added to the strainer containing viscous material. At this point, there are almost no visible pieces remaining in the tube. A syringe plunger is used to disrupt the pieces through the strainer, followed by washing with the flow-through medium until the strainer is clean. The flow-through is collected in the same 50 ml tube on ice (Fraction 3). Fractions -1, -2 and -3 are centrifuged for 6 minutes at 1200 rpm, 4°C and then resuspended in 5 ml of PBS/EDTA. The fractions are pooled and centrifuged for 10 min at 1200 rpm, 4°C.

To separate the leukocytes from the mouse oral epithelial cells (MOEC), connective tissues, dead cells and tissue clumps, the cells are resuspended in 4 ml of 30% Percoll (made in PBS/EDTA) in a 15 ml tube. Using Pasteur pipettes, 5 ml of 70% Percoll (made in PBS/EDTA), is carefully layered under the 4 ml cell suspension in a 15 ml tube. The tube is centrifuged for 15 min at 1100 × g, room temperature, with a low acceleration rate. The fraction of cells enriched in epithelial cells float on the 30% Percoll layer, while leukocytes, including intra-epithelial and lamina propria immune cells are found between the 30% and 70% layer. We refer to this population as mouse oral intra-epithelial and lamina propria leukocytes (MOIL). Debris and dead cells will pellet at the bottom of the conical tube. MOEC are removed carefully and can be further purified if relevant. MOIL are carefully collected, resuspended in complete RPMI-1640 with 10% FBS in a 15 ml tube and washed twice before use for cultures or immunofluorescence staining.

**Figure 1:**
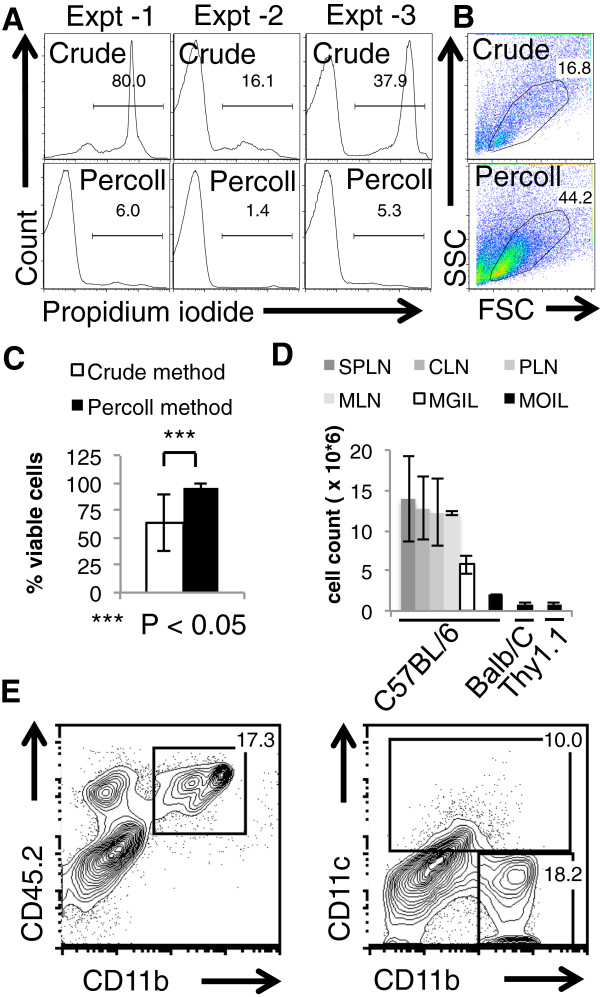
**Viability, recovery and the expression of CD45, CD11c and CD11b in mouse oral intra-epithelial and lamina propria leukocytes (MOIL) isolated from the oral tissues of mice.** Flow cytometric histograms showing propidium iodide (PI) uptake by the dead cells **(A)**, or dot plots showing conventional leukocyte gating in our flow cytometry analyses **(B)**, in crude cell preparations (top) and those isolated by percoll separation (bottom). **(C)** The percentage of viable cells (PI negative), as assessed by flow cytometry as in **(A)**. **(D)**, MOIL cell recovery (cell count/1 mouse) from various strains of mice, as enumerated using hemocytometer. **(E)** Flow cytometric contour plots showing CD45.2, CD11b and CD11c expression, which are the hematopoietic, monocytic, and dendritic cell markers, respectively. Percentages of marker expressing MOIL (pooled from 2 mice) are shown in the gates. All these data represent at least three to five independent experiments using at least 10- 15 mice. P values were calculated by Students ‘t’ test in Microsoft Excel software using two tailed distribution and two-sample equal variance parameters.

**Figure 2:**
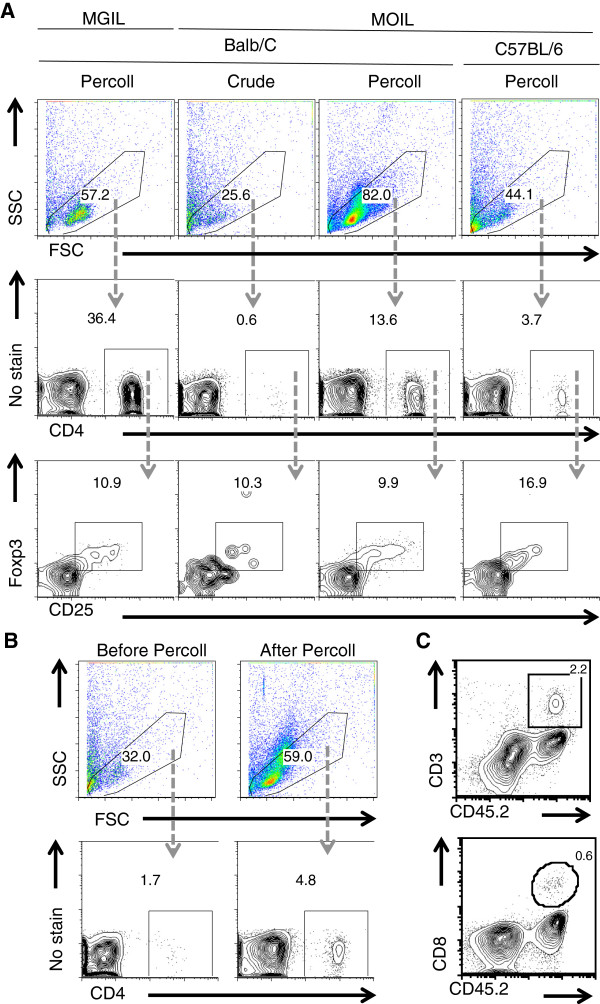
**Leukocyte and CD4 cell enrichment in MOIL isolated from the oral tissues using our method. (A)** Flow cytometric dot plots showing leukocyte gates (top), CD4 expression (middle) and CD25 and Foxp3 expression (bottom) in MOIL isolated by different methods (pooled from 10 mice) from different strains of mice (gated as shown). Mouse gut intra-epithelial and lamina propria leukocytes (MGIL) are shown as a control. **(B)** Flow cytometric dot plots showing leukocyte gates (top) and CD4 expression (bottom) before (left) and after (right) the percoll centrifugation **(C)** Contour plots showing CD45.2, CD3 and CD8 expression, which are the hematopoietic, T lymphocytic, and CD8+ T lymphocytic markers, respectively. Percentages of marker expressing cells are shown in the gates. All these data represent at least three to five independent experiments using at least 10- 15 mice.

**Figure 3:**
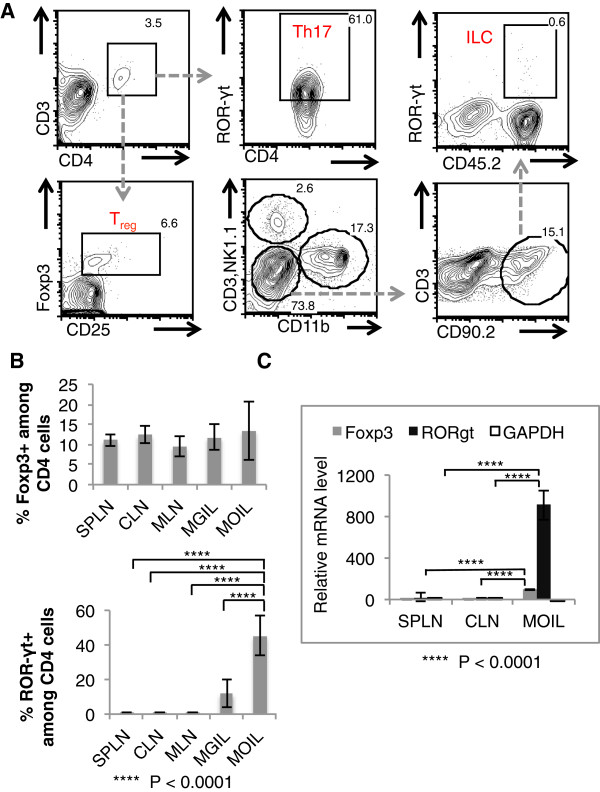
**Specialized T cell subsets in MOIL isolated from the oral tissues of C57BL/6 mice. (A)** Flow cytometric contour plots showing the expression of various immune cell markers in MOIL cells CD3+ CD4+ RORγt + cells are shown as Th17 cells, CD3 + CD4 + CD25 + Foxp3+ cells are shown as T_regs_, CD11b- NK1.1- CD3- CD90.2+ CD45.2+ RORγt are shown as innate lymphoid cells (ILC). Percentages of marker expressing cells are shown in the gates. These data are representative of two independent experiments using cells pooled from 10 mice. **(B)** Percentage of CD4 + Foxp3 (top) and CD4+ RORγt (bottom), in MOIL as assessed by flow cytometry from 5 independent experiments (as in Figure 2A). Spleen (SPLN), cervical lymph nodes (CLN), mesenteric lymph nodes (MLN) and MGIL are used as controls. **(C)** Real time quantitative PCR analyses of mRNA levels of Foxp3, RORγt and GAPDH (house keeping gene control) in cells isolated from SPLN, CLN and MOIL, with SPLN normalized to “1” to calculate the relative mRNA levels. These data are representative of three independent experiments using 15 mice. P values were calculated by Ordinary one-way ANOVA test, with multiple comparisons, using Prism 6.0 (GraphPad Software, Inc.).

**Figure 4:**
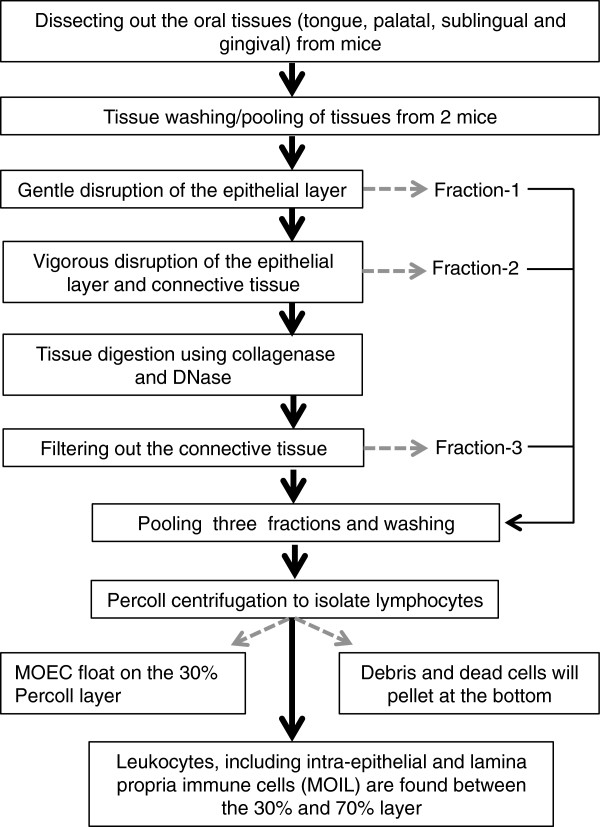
Flow chart of the major steps in the MOIL isolation method.

Consistently, as shown by the viable stain propidium iodide, the viability of the MOIL that we isolated was > 94% (propidium iodide negative) (Figure 1A, bottom, 1C). The crude method that just disrupts the tissue using EDTA and enzymatic digestion, yields cells that show inconsistent viability and recovery (Figure 1A, top, 1C). In Figure 1A, we used only the conventional leukocyte gate in the forward scatter/side scatter dot plot, as in all our analyses (unless otherwise specified) (Figure 1B). We obtained ~ 0.8 × 10^6^ – 1.2 × 10^6^ MOIL from the oral tissue of one mouse *ex vivo* from mouse oral tissues, compared to 5 - 6 × 10^6^ mouse gut intra-epithelial and lamina propria leukocytes (MGIL), isolated from the colon using a similar method (Figure 1D). In this population of MOIL, we detected the presence of CD45+ hematopoietic cells, CD11c + dendritic cells, and CD11b + monocytes/macrophages (Figure 1E). Most importantly, using our method, we could better enrich the leukocytes (up to 80%) (Figure 2A, top), CD4+ T cells (up to 13%) (Figure 2A, middle) compared to the crude method, which much lower percentages of leukocytes. We found that the frequency of CD4+ T cells is higher in Balb/C mice than C57BL/6 mice in oral tissues (Figure 2A, middle). Although the frequency of CD4 cells in MOIL (3- 13%) is not as high as in MGIL (10 - 30%), we could detect these cells and CD25 + Foxp3+ cells consistently (Figure 2A, bottom). The percoll centrifugation especially enriches the leukocyte fraction (Figure 2B, top) in general, and also the CD4+ cells (Figure 2B, bottom) among the isolated MOIL. Moreover we could also detect CD3+ CD45+ hematopoetic T cells that also included CD8+ T cells, (Figure 2C) and CD4+ cells as well (Figure 3A). Among the CD3 + CD4+ cells, we found CD25 + Foxp3+ T_regs_ at a frequency of 6 - 17%, and CD44^high^, ROR-γt + Th17 cells up to a frequency of 40-60% in MOIL from C57BL/6 mice (Figure 3A). We also detected the presence of recently discovered CD90.2+ ROR-γt + CD3- NK1.1- CD19- group 3 innate lymphoid cells (ILC) among MOIL (Figure 3A). Flow cytometry data from at least 5 experiments show that the frequency of CD4 + CD25 + Foxp3+ T_regs_ is slightly higher in MOIL preparations, compared to other tissue and lymphoid organs we examined (Figure 2A, bottom, Figure 3B). Moreover, ROR-γt expressing CD4+ cells (Th17 cells) are significantly enriched in the MOIL preparations compared to other tissue and lymphoid organs (Figure 3B). These data were also confirmed by real-time quantitative PCR (Figure 3C). The cells isolated are ~95% viable and can be further purified using cell sorting techniques and cultured *in vitro* to study their precise functions [16]. The major steps of the protocol are outlined in a flow chart (Figure 4).

This method will permit us to investigate the oral tissue immune cells more carefully. We will employ this method to study immune cell interactions with microbes in infection models such as oropharyngeal candidiasis (OPC) in mice. An important advantage of studying immune mechanisms in the oral cavity at the cellular level is that it will lead to novel ways of non-invasive mucosal immunotherapy in easily accessible oral mucosal tissue, compared to other sites such as gut and rectal mucosa.

## Abbreviations

MOIL: Mouse oral intraepithelial and lamina propria immune cells; PBS: Phosphate buffered saline; RPMI: Roswell park memorial institute; EDTA: Ethylenediaminetetraacetic acid; FBS: Fetal bovine serum; DTT: Dithiothreitol; OPC: Oropharyngeal candidiasis.

## Competing interests

The authors declare that they have no competing interests.

## Authors’ contributions

PP conceived, designed, and performed replicate experiments; acquired, analyzed and interpreted the data; wrote and revised the manuscript. AW revised the manuscript critically for important intellectual content. NB and YZ were involved in performing the isolation procedures. All authors read and approved the final manuscript.
